# Lethal versus surviving sepsis phenotypes displayed a partly differential regional expression of neurotransmitters and inflammation and did not modify the blood–brain barrier permeability in female CLP mice

**DOI:** 10.1186/s40635-024-00688-7

**Published:** 2024-11-04

**Authors:** Fatemeh Azizian-Farsani, Katrin Weixelbaumer, Daniel Mascher, Andrea Klang, Sandra Högler, Nora Dinhopl, Barbara Bauder, Herbert Weissenböck, Alexander Tichy, Peter Schmidt, Hermann Mascher, Marcin F. Osuchowski

**Affiliations:** 1 Present Address: Ludwig Boltzmann Institute for Traumatology, The Research Center in Cooperation with AUVA, Donaueschingenstraße 13, 1200 Vienna, Austria; 2 pharm-analyt Labor GmbH, Baden, Austria; 3 Institute of Pathology, University of Veterinary Medicine, Vienna, Austria; 4 Institute of Medical Physics and Biostatistics, University of Veterinary Medicine, Vienna, Austria

**Keywords:** Abdominal sepsis, Brain amines, Cytokines, Phenotyping, Outcome prediction

## Abstract

**Background:**

Septic encephalopathy is frequent but its pathophysiology is enigmatic. We studied expression of neurotransmitters, inflammation and integrity of the blood–brain barrier (BBB) in several brain regions during abdominal sepsis. We compared mice with either lethal or surviving phenotype in the first 4 sepsis days. Mature CD-1 females underwent cecal ligation and puncture (CLP). Body temperature (BT) was measured daily and predicted-to-die (within 24 h) mice (for P-DIE; BT < 28 °C) were sacrificed together (1:1 ratio) with mice predicted-to-survive (P-SUR; BT > 35 °C), and healthy controls (CON). Brains were dissected into neocortex, cerebellum, midbrain, medulla, striatum, hypothalamus and hippocampus.

**Results:**

CLP mice showed an up to threefold rise of serotonin in the hippocampus, 5-hydroxyindoleacetic and homovanillic acid (HVA) in nearly all regions vs. CON. Compared to P-SUR, P-DIE mice showed a 1.7 to twofold rise of HVA (386 ng/g of tissue), dopamine (265 ng/g) and 3,4-Dihydroxyphenylacetic acid (DOPAC; 140 ng/g) in the hippocampus, hypothalamus and medulla (174, 156, 82 ng/g of tissue, respectively). CLP increased expression of TNFα, IL-1β and IL-6 mRNA by several folds in the midbrain, cerebellum and hippocampus versus CON. The same cytokines were further elevated in P-DIE vs P-SUR in the midbrain and cerebellum. Activation of astrocytes and microglia was robust across regions but remained typically phenotype independent. There was a similar influx of sodium fluorescein across the BBB in both P-DIE and P-SUR mice.

**Conclusions:**

Compared to survivors, the lethal phenotype induced a stronger deregulation of amine metabolism and cytokine expression in selected brain regions, but the BBB permeability remained similar regardless of the predicted outcome.

**Supplementary Information:**

The online version contains supplementary material available at 10.1186/s40635-024-00688-7.

## Introduction

Complex pathophysiological dynamics occurring in sepsis syndromes typically involve multiple organ systems of the body often resulting in multiple-organ dysfunction and/or subsequent death [1]. The nervous system, with its central and peripheral components, is one of the most heavily affected systems by sepsis syndromes [2, 3]. This is exemplified in both Sequential Organ Failure Assessment (SOFA) and quick (q)SOFA scores that integrate a neurologic assessment. The nervous system disturbances are clinically diagnosed as sepsis-associated encephalopathy (SAE), sepsis-associated delirium (SAD) [4], critical illness polyneuropathy (CIP) and myopathy (CIM) [5]. For example, SAE has been reported to be one of the most common causes of delirium in the intensive care unit (ICU) patients [6] and delirium is found in up to 87% of cases [7]. This wide range is partly attributed to the fact that in heavily instrumented and/or sedated septic patients, central nervous system (CNS)-related disturbances are frequently underdiagnosed. Severity of SAE varies from the lack of attention and confusion in mild cases to hypoactivity and coma; presence of the latter two has been directly associated with mortality [8]. Sepsis survivors often display long-term cognitive impairments indicating a chronic brain damage despite recovery from the initial septic insult [9]. Numerous mechanisms triggered by sepsis have been proposed to underlie SAE, yet its ontogeny is complex and remains enigmatic.

Intuitively, altered neurotransmitter expression and/or metabolism are speculated to underlie clinical manifestations of cerebral dysfunction in sepsis. For example, hyperactivity of the dopaminergic and noradrenergic systems was plausibly linked to the presence of delirium already two decades ago [10]. This is clinically supported by the fact that dopamine antagonists are used for the treatment of hyperactive delirium and anti-depressants with dopaminergic activity are associated with delirium [11, 12]. More recently, decreased dopamine, changes in serotonin activity or relative serotonin deficiency have been associated with hepatic encephalopathy [13, 14]. A general assumption that increased availability of brain amines can promote delirium is also suggested given that the LPS administration to healthy volunteers increased the brain influx of phenylalanine (norepinephrine substrate) [15]. Similarly, deranged amino acid ratios were detected in acutely febrile elderly patients [16] and altered tryptophan/large neutral amino acids were associated with delirium in the ICU [17, 18]. While the existing studies support the notion of deregulated neuronal function in sepsis, clear-cut evidence of pathophysiological derangements in the CNS, their grade, localized dynamics and their association with sepsis outcomes are lacking.

Sepsis-induced disruptions in neurotransmitter homeostasis result from multifactorial systemic perturbations. Early interplay between systemic immunity and the CNS precipitates neuronal dysfunction in SAE [19]. The initiation of sepsis-triggered systemic and localized brain inflammation, characterized by inflammatory mediator release, astrocytic dysfunction, microglial activation, blood–brain barrier (BBB) disruption, diminished cerebral blood flow, and cerebral edema, collectively impact CNS functionality [20, 21]. Inflammatory cytokine release in sepsis alters brain amines and neuronal signaling pathways, exerting effects both locally and systemically [21, 22]. Neuroinflammation is implicated in chronic neurodegenerative disorders [23] and acute CNS injury [24]. Cytokines contribute to sepsis-induced neurodeficits in diverse models, including cecal ligation and puncture (CLP) and intraperitoneal injection of cecal material [25–29].

In this mechanistic study, we characterized the regional brain amine concentrations and cross-matched it with the local inflammatory activity and BBB integrity in septic mice that displayed either a lethal or surviving disease phenotype in the acute phase of polymicrobial abdominal sepsis.

## Materials and methods

### Animals

Three-month-old female (average weight 30 g), outbred HSD: ICR mice (CD-1; Harlan Laboratories) were used for all experiments. Mice were housed in groups of five animals per cage (type III, high) with a 12 h light/dark cycle, controlled temperature (21–23 °C) and humidity (50 ± 2.5%). All mice were housed in an isolated and relatively small animal room with quick access that allows frequent monitoring. Bi-weekly cage changes and water changes were performed. Standard rodent feed and drinking water were offered ad libitum. Cages were enriched with houses, wood wool for nesting as well as wooden boards, tunnels and small blocks for gnawing (Abedd Lab & Vet Service, Austria) to facilitate natural behavior prior to and throughout the experiments. All animal procedures were approved by the Viennese (Austrian) legislative committee (authorization no: 0007602/2007/10) and conducted according to National Institute of Health guidelines.

### Polymicrobial sepsis model

Mice were subjected to CLP surgery to induce polymicrobial abdominal sepsis. Procedures were conducted following the original protocol by Wichterman et al*.* [30] with modifications specified elsewhere [31, 32] and adhered to the MQTIPSS guidelines whenever feasible [33]. Briefly, mice were anesthetized with Isoflurane (Forane^®^) and after opening the abdominal cavity via midline laparotomy, the cecum was exposed, ligated directly under the ileocecal valve and punctured twice with a 17-gauge needle to achieve between 30 and 50% mortality 5 days after surgery. The abdominal wall was closed with 2 single button sutures and skin adapted with Histoacryl^®^ adhesive. Mice received wide-range antibiotic therapy (25 mg/kg imipenem, Zienam^®^) and fluid substitution (1 mL Ringer’s solution) with an analgesic (0.05 mg/kg buprenorphine, Buprenovet^®^) twice daily for 5 consecutive days post-CLP. While the study has is predominantly mechanistic, the above treatments were employed to characterize the CLP responses in an environment approaching a clinical reality. Control animals did not receive any surgical manipulation and/or treatment.

### Prediction of CLP outcome based on body temperature

All mice were monitored by at least 3 ×/day and more frequently whenever a mouse condition worsened. To avoid/reduce unnecessary suffering of CLP mice facing imminent death, we use in our laboratory a custom-developed scoring approach that combines the mouse clinical assessment scoring system (M-CASS) [34] and sequential BT measurements. The combination of M-CASS and BT monitoring employed for prediction of early outcome (days 1–4 post-CLP) allows a proper balance between humane treatment and experimental rigor in sepsis modeling [35]. In brief, rectal BT measurements using the Fluke 52 Series II thermometer (Fluke, USA) and well-being of mice were assessed every 6–12 h (starting 12 h post-CLP). Mice were euthanized once the score indicated imminent death (score ≥ 8 and/or BT < 28 °C as detailed elsewhere [31]. A separate group of healthy control mice (no CLP) was also sacrificed as reference. The M-CASS/BT-based prediction of outcome we developed in our laboratory is very precise (AUC = 0.94) [32]. This predictive system prevents an over-liberal euthanasia decision-making, that is, allocation of potential survivors to the P-DIE group in survival studies. While the disparity in the BT dynamic between septic mice and humans is acknowledged [36], hypothermia constitutes an important predictor of mortality in septic patients [37–40]. In mice, deep hypothermia signals severe immuno-dysfunction, aiding in the study of lethal sepsis phenotypes [41, 42].

### Sacrifice scheme for P-DIE/P-SUR mice

Mice were always sacrificed in pairs: i.e., each mouse identified as P-DIE (lethal phenotype; score ≥ 8 and/or BT < 28 °C) was sacrificed together with a mouse identified as P-SUR (surviving phenotype; score ≤ 3 and/or BT > 35 °C) and the brains were harvested for analysis. All the mouse pairs were sacrificed between days 2 and 4 after CLP. The septic pairs were typically accompanied by the healthy control mouse. There was the following distribution of P-DIE/P-SUR pairs. In the pairs allocated for neurotransmitter and mRNA analysis: Day 2 post-CLP: 4 pairs; Day 3: 3 pairs; Day 4: 1 pair. Total of 8 pairs. In the pairs allocated for BBB and immunohistochemistry (IHC) assays: Day 2 post-CLP: 4 pairs, Day 3: 4 pairs; Day 4: 1 pair (but only P-SUR mouse was included as P-DIE failed to correctly perfuse). Total of 8 pairs + 1 extra P-SUR mouse. For neurotransmitters and cytokines, 3–8 P-SUR/P-DIE pairs (depending on the part of the brain—view figure's legend for details) and 3–7 control mice were sacrificed. For IHC and BBB integrity, 8 P-SUR/P-DIE pairs and 3 control mice were sacrificed.

### Brain dissection

All mice were subjected to isoflurane anaesthesia and were exsanguinated by transcardiac perfusion with heparinized NaCl. Next, the brain was removed from the scull and cut along the sagittal plane into the two hemispheres. Each hemisphere was dissected according to the protocol of Glowinski and Iversen [43] with slight modifications. Specifically, each brain was dissected into seven regions: (1) neocortex, (2) midbrain, (3) hypothalamus, (4) hippocampus, (5) striatum, (6) cerebellum and (7) medulla (oblongata). After removing the brain from the skull, the brain regions dissected from the left hemisphere were placed into ice-cold 0.05 M perchloric acid with 0.1% cystein in tared vials. Each sample was gently homogenized by a portable homogenizer (IKA-Werke GmbH & Co. KG) in the vial placed on ice and subsequently centrifuged at 12,000 g for 5 min at 4 °C as previously described (see “Neurotransmitter analysis”) [44]. Brain regions from the right brain half were immediately snap-frozen in liquid nitrogen and stored at − 80 °C for analysis of cytokine mRNA expression (see Gene expression). From a second cohort of CLP mice, the right brain half was separated into three regions: (1) neocortex, (2) cerebellum and (3) midbrain to investigate the BBB integrity, which was performed on brain homogenate after intravenous fluorescein injection (see “Fluorescein permeability” below) and the other half was processed for immunohistochemical examinations.

### Gene expression

Total RNA from the dissected brain regions was extracted using peqGOLD TriFast (Peqlab, Germany). Specifically, RNA was extracted by adding 400 µL chloroform, shaking for 15 s. by hand followed by centrifugation at 4 °C, 12.000×*g* for 15 min. The upper clear phase was transferred to a new tube and precipitated by adding 500 µL 2-Propanol. After mixing and incubation, tube was centrifuged at 4 °C, 12,000×*g* for 10 min. The pellet washed with 1 mL 70% Ethanol by centrifuging at 4 °C 7500×*g* for 5 min. Supernatant was discharged and pellet air dried for 5–10 min, then resuspended in ddH_2_O. Yield (260 nm) and purity (260 nm/280 nm) of the isolated RNA were checked by spectrophotometry and the RNA integrity was confirmed by agarose gel electrophoresis. 2 μg of the total RNA were treated with DNase I (Promega, USA) and used for complementary DNA (cDNA) synthesis using anchored oligodT18-primers (Microsynth, Swizerland) and AMV Reverse Transcriptase (Finnzymes, Finland).

Gene expression of the typically sepsis-measured cytokines [45] including murine interleukin-6 (IL-6), tumor necrosis factor alpha (TNFα), interleukin-1β (IL-1β), monocyte chemotactic protein-1 (MCP-1), transforming growth factor-β1 (TGF-β1), interleukin-1 receptor antagonist (IL-1ra) and hypoxanthine–guanine phosphoribosyltransferase (HPRT) was analyzed by real-time polymerase chain reaction (Real time PCR) on a CFX96 real-time cycler (Bio-Rad, Austria) using KAPA SYBR Fast Universal 2× Mastermix (Peqlab, Germany). Following protocol was used: (1) 95 °C 3 min, (2) 95 °C 10 s., (3) Ta of the specific primer for 30 s, (4) 72 °C 10 s, (5) GO TO (2.) 39×, (6) 95 °C 10 s, (7) Ta 30 s, (8) melting from Ta to 95 °C in 0.5 °C for 5 s steps. Following primers were used: HPRT sense (s) 5ʹ-GCAAGTCTTTCAGTCCTGTCC-3ʹ and antisense (anti-s) 5ʹ-GCAGCGTTTCTGAGCCAT-3ʹ Ta(exp.): 60 °C, Tm(exp.): 87.5 °C; TGFß1 s: GCTACCATGCCAACTTCTGT, anti-s: CGTAGTAHACGATGGGCAGT Ta(exp.): 58 °C, Tm(exp.): 86 °C; IL-1ra s: CCAAACACCATCTTCACTCC, and anti-s: GCAGAGGAAACAGTCAGGAA Ta(exp.): 60 °C, Tm(exp.): 79.5 °C; IL-1ß s: ATCTGCGACGAGGAAGAGAA, and anti-s: ATCGCAGATGAAGCTCTGGT Ta(exp.): 60 °C, Tm(exp.): 87.5 °C; TNFα s: GTTCTATGGCCCAGACCCTCACA, and anti-s: TCCCAGGTATATGGGTTCATACC Ta(exp.): 60 °C, Tm(exp.): 86 °C; IL-6s: TTCCATCCAGTTGCCTTCTT, and anti-s: CAGAATTGCCATTGCACAAC Ta(exp.): 60 °C, Tm(exp.): 81 °C; MCP-1 s: AGGTCCCTGTCATGCTTCTG, and anti-s: CAAGAAGGAATCGGTCCAGA Ta(exp.): 65 °C, Tm(exp.): 72.5 °C. Target expression relative to HPRT was calculated using the 2^−ΔCt^ method [46].

### Neurotransmitter analysis

All neurotransmitters were measured by a specific and highly sensitive liquid chromatography tandem mass spectrometry assay (LC–MS/MS at pharm-analyt laboratory GmbH, Baden, Austria). The following brain amines and their metabolites were analysed and quantified: (1) serotonine (5-HT), (2) dopamine (DA), (3) homovanillic acid (HVA), (4) norepinephrine (NE), (5) hydroxyindoleatic acid (HIAA) (6) dioxyphenylacetic acid (DOPAC). Additionally Dihydroxy-l-phenylalanine (l-DOPA) and methoxy-hydroxy-phenylethylen glycol (MHGP) were measured in a small sub-cohort of mice (supplementary Figs. 1 and 2). Specifically, homogenized brain samples were immediately centrifuged at 12000*g* for 5 min at 4 °C and supernatants were filtered using a 0.2 µm filter (Poretics, Livermore, USA) and the filtrates were stored in − 85 °C until analysis.

The lower limit of quantification (LLOQ) in 100 µL filtrate was about 0.3 ng for 5-HT, 0.4 ng for DA, 0.2 ng for HVA, 2 ng for NE, 0.2 ng for HIAA, 0.03 ng for VMA, 0.4 ng for VA, 0.2 ng for DOPAC, 1 ng for l-DOPA, 0.04 ng for 3-MT, and 0.2 ng for MHGP. HPLC–MS/MS detection was done with an API-5000 tandem-MS-system (ABSciex, USA); HPLC column used was a C8 column. HIAA, VMA, HVA, DOPAC, VA, and MHPG were determined in negative ion mode with ESI using 1% acetic acid and acetonitrile as mobile phase; 5-HT, DA, L-DOPA, NE, and 3-MT were determined in positive ion mode with ESI using nonafluoropentanoic acid as counter ion for chromatography.

### Fluorescein permeability

Sodium-fluorescein (NaFl) permeability assay was used to assess the functional integrity of the BBB in a separate group of CLP mice (and healthy controls) following the stratification approach described above and analytical protocol described elsewhere [47]. Each enrolled mouse received an injection of sodium-fluorescein (200 µL of 0.5%) or a similar volume of physiologically buffered saline (PBS) by tail vein injection and was sacrificed 30 min later (undergoing a transcardiac perfusion with PBS). To simplify the analysis, the brain was dissected into three regions: (1) neocortex (with hippocampus), (2) cerebellum, (3) midbrain (with striatum and hypothalamus). After dissection, each region was immediately placed into 100 µL of *n*-butanol, homogenized by ultra-sonication, centrifuged for 15 min at 2500 rpm and 50 µL of the supernatant diluted in 200 µL of borate buffer for spectrophotometric analysis of the fluorescein signal (Polarstar Omega B.M.G. Labtech, Allmendgrün 8, Ortenberg, Germany). The NaFl was employed since its small molecular weight of 376 daltons can reveal a relatively small disruption of the BBB permeability.

### Histopathological and immunohistochemical examination

For histopathologic and immunohistochemical examination, the right brain half was fixed in 4% buffered formalin, then embedded in paraffin-wax. Samples were sectioned (~ 3 µm) and stained with standard Hematoxylin–Eosin (HE) and Fluoro-Jade B^®^ [48] (Millipore, Vienna, Austria). Immunohistochemical staining was carried out using autostainer 360 (Lab Vision, Thermo Fisher Scientific, Bonn, Germany) with the primary antibodies against glial fibrillary acidic protein (GFAP), (polyclonal anti-rabbit, dilution 1:7000, DakoCytomation, Glostrup, Denmark) and ionized calcium binding adaptor molecule 1 (IBA-1), (polyclonal anti-rabbit, dilution 1:2000, Wako Chemicals GmbH, Neuss, Germany). Prior to the anti-GFAP staining, the tissue sections were proteolytically pre-treated with pronase for antigen unmasking. No antigen-retrieval procedure was necessary for IBA-1 detection. For streptavidin–biotin immunoenzymatic antigen detection, biotinylated goat anti-rabbit solution was applied according to the manufacturer’s instructions (Thermo Fisher Scientific, Bonn, Germany). All histopathologic and immunohistochemical brain samples were independently examined in a blinded fashion by a certified veterinary pathologist from the Institute of Pathology, Centre of Pathobiology, Department of Biological Sciences and Pathobiology, University of Veterinary Medicine, Vienna. Histological evaluation, specifically vacuolisation was semi quantitively scored as following: 0 = no vacuolization, 1 = mild vacuolization, 2 = moderate vacuolization, 3 = severe vacuolization. Immunohistochemical evaluation of Iba-1 and GFAP staining was semi-quantitively scored as following: 0 = no signal, 1 = mild signal, 1.5 = mild to moderate signal, 2 = moderate signal, 2.5 = moderate to severe signal, 3 = severe signal.

### Electron microscopy (ELMI)

For electron microscopy, selected brain tissue samples were fixed in 5% glutaraldehyde (Merck, Darmstadt, Germany) in 0.1 M phosphate buffer (Sigma–Aldrich, Vienna, Austria), pH 7.2, at 4 °C for 3 h. Subsequently, samples were post-fixed in 1% osmium tetroxide (Merck) in the same buffer at 4 °C for 2 h. After dehydration in an alcohol gradient series and propylene oxide (Merck), the tissue samples were embedded in glycid ether 100 (Serva, Heidelberg, Germany). Ultrathin sections were cut on a Leica ultramicrotome (Leica Ultracut S, Vienna, Austria), stained with uranyl acetate (Sigma–Aldrich) and lead citrate (Merck) and examined with a Zeiss TEM 900 electron microscope (Carl Zeiss, Oberkochen, Germany) operated at 60 kV. All ELMI samples were examined by a veterinary pathologist in a blinded fashion from the Institute of Pathology, Centre of Pathobiology, Department of Biological Sciences and Pathobiology, University of Veterinary Medicine, Vienna.

### Statistical analysis

All quantitative data were tested for normality and non-Gaussian data sets were log-transformed and re-tested for normality before further statistical analysis. All comparisons between septic mice (regardless of the predicted outcome) and CON were carried out by Student *t* test with Welch’s correction whenever necessary and Mann–Whitney test. Subgroup comparisons among P-DIE and P-SUR were carried out using either one-way ANOVA with Bonferroni (parametric data) or Kruskal–Wallis with Dunn’s post hoc test (non-parametric data) for selected pairs (including adjustments for repeated measurements). Statistical tests for data presented in the figures were carried out using Prism 5 (GraphPad Software Inc., San Diego, CA, USA). Statistical analysis of histopathological changes shown in the Tables (Tables 1 and 2, Suppl. Table 1) was performed using IBM SPSS v.19 (Armonk, NY, USA). Differences between groups within each brain region were analysed using Mann–Whitney *U* test. All quantitative data are presented on the normal scale with mean ± standard deviation unless noted otherwise. The level of *p* < 0.05 was considered significant. As TEM displays a technique which is focused on selected regions on the ultrastructural level and was performed on selected samples, statistical verification was not performed.

**Table 1:** GFAP expression of all evaluated brain regions

Brain region	CON	P-SUR	P-DIE	CON/P-SUR	CON/P-DIE	P-SUR/P-DIE	CON/SEPSIS
	*n* = 3	*n* = 8	*n* = 8	*p*-value	*p*-value	*p*-value	*p*-value
Neocortex	1.0 (0.5–1.0)	1.3 (1.0–1.5)	1.3 (1.0–1.5)	0.07	0.07	0.81	0.05
Cerebellum	1.0 (1.0–1.0)	3.0 (2.5–3.0)	3.0 (2.1–3.0)	0.01	0.01	0.86	0.00
Medulla oblongata	1.0 (1.0–1.0)	2.0 (2.0–2.5)	2.8 (2.1–3.0)	0.01	0.01	0.04*	0.01
Hypothalamus	1.5 (1.0–1.5)	2.0 (2.0–2.0)	2.0 (2.0–2.0)	0.03	0.00	0.59	0.00
Midbrain	1.0 (1.0–1.0)	1.5 (1.5–2.0)	1.5 (1.5–2.0)	0.03	0.08	0.77	0.03
Hippocampus	2.0 (2.0–2.0)	2.5 (2.1–3.0)	3.0 (2.3–3.0)	0.05	0.03	0.29	0.03
Striatum	1.0 (0.5–1.0)	1.3 (1.0–1.5)	1.5 (1.0–1.9)	0.07	0.05	0.53	0.04

^*^p < 0.05 in P-SUR vs P-DIE by Mann–Whitney-*U*-test (with an adjustment for multiple comparisons). Each region/mouse was scanned for staining intensity and results were tallied based on the group allocation and compared. 0 = no signal, 1 = mild signal, 1.5 = mild to moderate signal, 2 = moderate signal, 2.5 = moderate to severe signal, 3 severe signal

**Table 2:** IBA-1 expression of all evaluated brain regions

Brain region	CON	P-SUR	P-DIE	CON/P-SUR	CON/P-DIE	P-SUR/P-DIE	CON/SEPSIS
	*n* = 3	*n* = 8	*n* = 8	*p*-value	*p*-value	*p*-value	*p*-value
Neocortex	1.0 (1.0–1.0)	2.0 (1.6–2.0)	1.8 (1.0–2.4)	0.02	0.09	0.58	0.03
Cerebellum	0.5 (0.5–0.5)	1.5 (1.5–2.0)	1.5 (1.1–1.9)	0.01	0.02	0.32	0.01
Medulla oblongata	1.0 (1.0–1.0)	2.0 (1.6–2.0)	1.8 (1.1–2.0)	0.02	0.05	0.56	0.02
Hypothalamus	1.0 (1.0–1.0)	1.5 (1.0–2.0)	1.5 (1.0–2.0)	0.14	0.14	1.00	0.12
Midbrain	1.0 (1.0–1.0)	2.0 (1.5–2.0)	1.8 (1.0–2.4)	0.02	0.09	0.87	0.03
Hippocampus	1.0 (1.0–1.0)	2.0 (1.0–2.5)	2.0 (1.1–2.5)	0.28	0.21	0.74	0.2
Striatum	2.0 (2.0–2.0)	2.0 (1.6–2.4)	1.8 (1.5–2.0)	1.00	0.31	0.44	0.58

Each region/mouse was scanned for staining intensity and results were tallied based on the group allocation and compared. *p* < 0.05 by Mann–Whitney-*U*-test (with an adjustment for multiple comparisons). 0 = no signal, 1 = mild signal, 1.5 = mild to moderate signal, 2 = moderate signal, 2.5 = moderate to severe signal, 3 severe signal

## Results

### CLP phenotypes differentially modulated regional neurotransmitter metabolism

To characterize the dynamics of the brain amine metabolism in the CNS during acute sepsis, the tissue concentration of three main neurotransmitters (and their main metabolites) was measured with HPLC in seven specific brain regions of CLP mice.

### CLP *sepsis* versus healthy

**Fig. 1:**
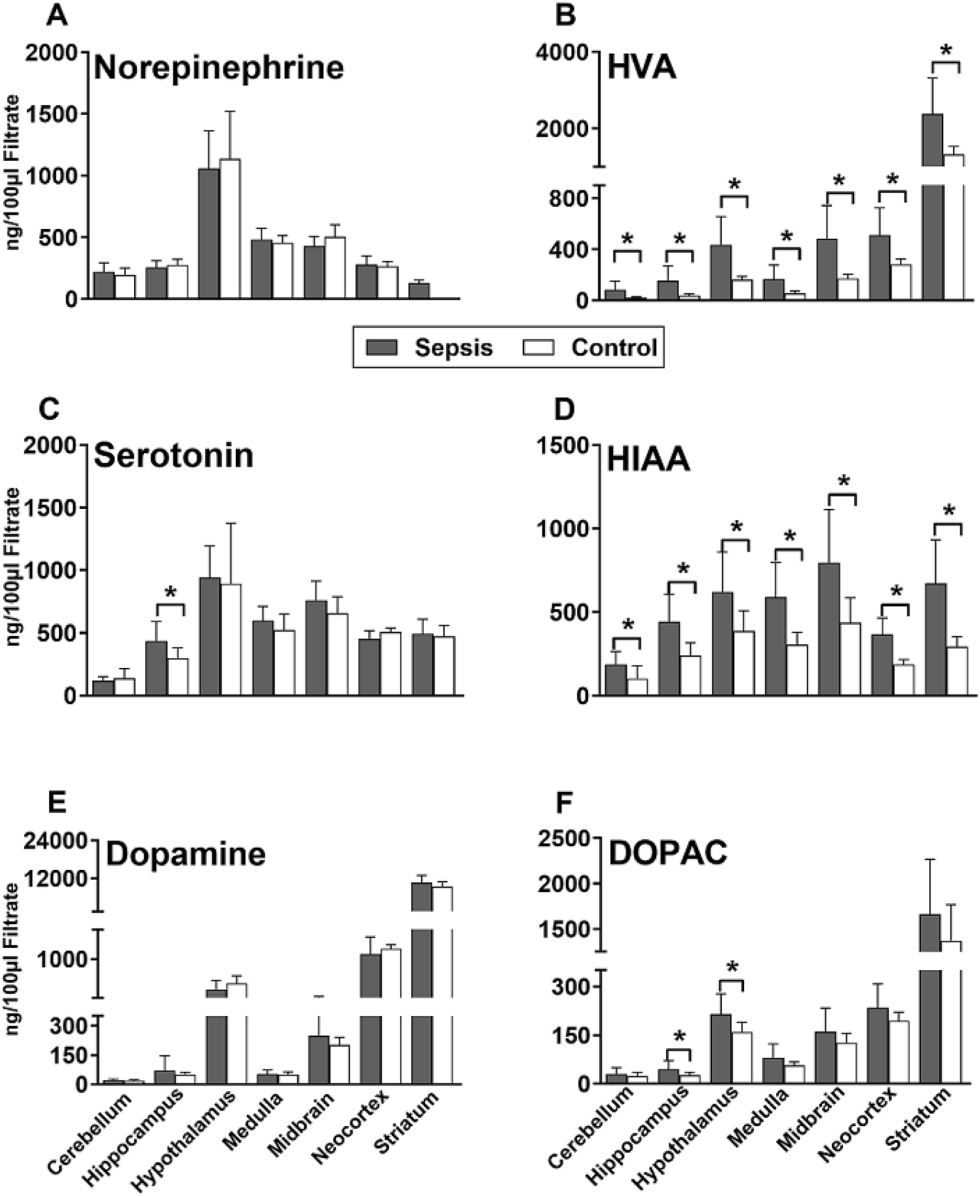
Regional concentrations of neurotransmitters and their selected metabolites (**A**–**F**) in the brains of septic (CLP) and healthy control mice. Septic mouse data were pooled regardless of the predicted outcome. Mice were sacrificed within days 2–4 post-CLP. CLP mice: *n* = 15–16 in all regions except neocortex (*n* = 10) and striatum (*n* = 7–10). Control mice: *n* = 7 in all regions except neocortex (*n* = 4) and striatum (*n* = 3). Data as mean ± SD. *P < 0.05. *HVA* homovanillic acid, *HIAA* hydroxyindoleacetic acid, *DOPAC* dihydroxyphenylacetic acid

First, regardless of the predicted outcome, data from all septic mice were pooled for comparisons (i.e. P-DIE and P-SUR mice combined) and compared to healthy animals. Early sepsis did not modulate the concentration of norepinephrine, serotonin and dopamine: their levels typically remained comparable to control (Fig. 1A, C, E). Only approx. 1.5 fold serotonin increase was shown in the hippocampus of septic mice (Fig. 1C).

In contrast, CLP increased the production of all neurotransmitter metabolites. HIAA (serotonin metabolite) and HVA (major catecholamine metabolite) were elevated after CLP in all seven brain regions compared to controls (Fig. 1B, D). The most pronounced HVA increase was in the hippocampus (fourfold increase), while HIAA peaked in the striatum (> twofold). Another dopamine metabolite, DOPAC increased in the hippocampus and hypothalamus (Fig. 1F).

In a smaller set of mice, we also analysed concentration of L-DOPA (the main catecholamine precursor) and MHPG (the principal norepinephrine metabolite). After CLP (compared to control), L-DOPA remained unaltered (Supplementary Fig. 1A), while MHPG increased in four regions (except hippocampus, Supplementary Fig. 1B).

### Septic mice P-DIE versus P-SUR

**Fig. 2:**
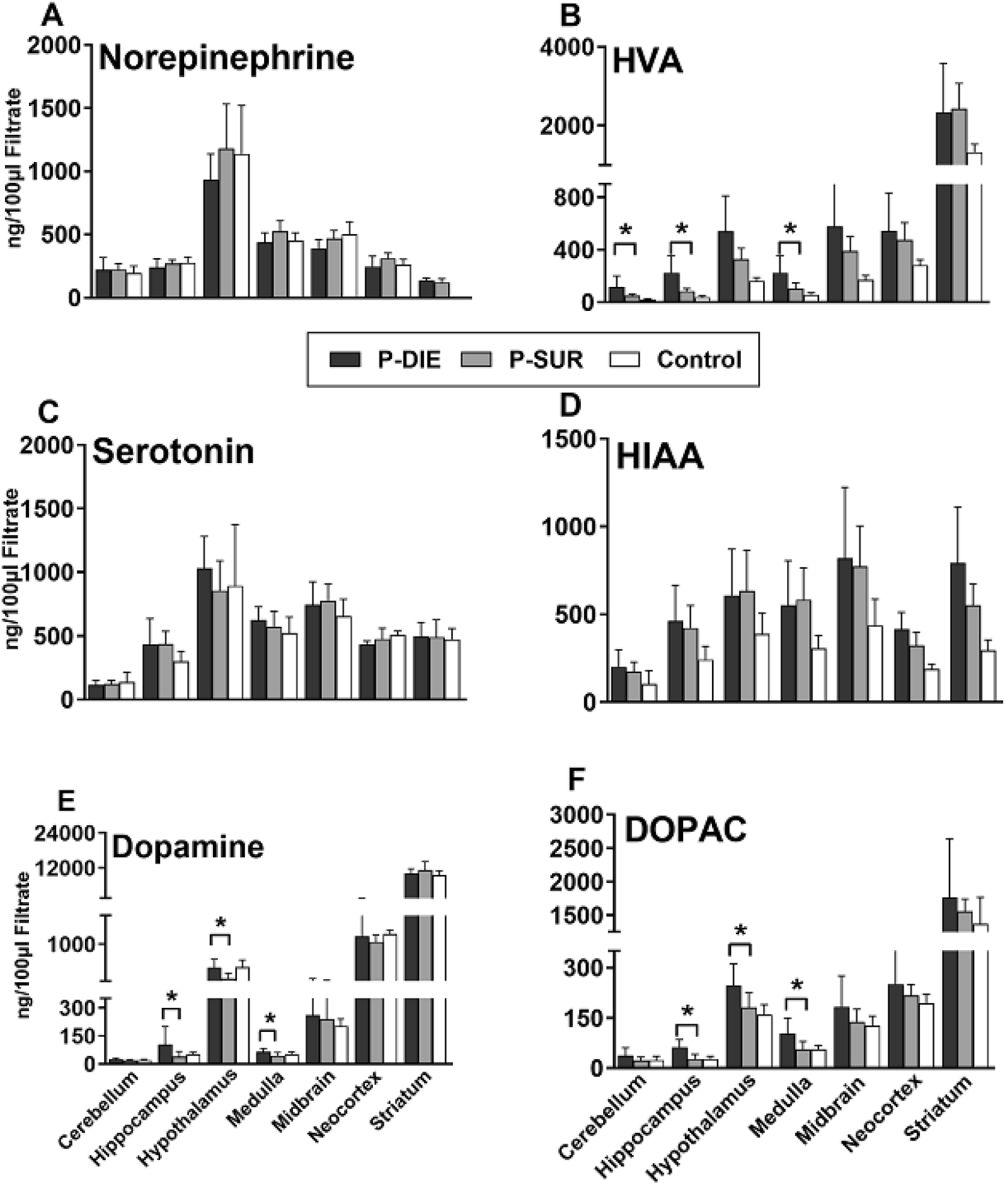
Regional concentrations of neurotransmitters and their selected metabolites (**A**–**F**) in the brains of septic (CLP) mice predicted-to-die (P-DIE), predicted-to-survive (P-SUR) and healthy control mice. P-DIE mice were sacrificed within days 2–4 post-CLP and matched with P-SUR mice. P-DIE: *n* = 7–8 in all regions except neocortex (n = 5) and striatum (n = 3). P-SUR: *n* = 7–8 in all regions except neocortex (*n* = 5) and striatum (*n* = 4) Control mice: *n* = 7 in all regions except neocortex (*n* = 4) and striatum (*n* = 3). Data as mean ± SD. *P < 0.05 between P-DIE and P-SUR. *HVA* homovanillic acid, *HIAA* hydroxyindoleacetic acid; DOPAC: dihydroxyphenylacetic acid

Next, we determined the outcome-dependent differences in the same targets: CLP mice were divided into P-DIE and P-SUR and the regional neurotransmitter concentrations were compared (Fig. 2). Compared to P-SUR, HVA, dopamine and DOPAC (but not norepinephrine, serotonin and HIAA) were elevated in several brain regions of P-DIE mice. HVA increased up to 2.7-fold in the cerebellum, hippocampus, hypothalamus and medulla, while dopamine and DOPAC in P-DIE mice robustly increased in the hippocampus (by 2.3 and 2.6-fold, respectively).

There were no changes between P-DIE and P-SUR mice in the L-DOPA and MHPG concentration in any of the analysed brain regions (Supplementary Fig. 2).

### CLP *sepsis* selectively activated regional cytokine gene expression in the brain

To precisely characterize regional cytokine responses in acute sepsis, gene expression of six key pro/anti-inflammatory cytokines was determined in the midbrain, cerebellum, hippocampus and hypothalamus. First, to estimate the general impact of sepsis, all septic mice (regardless of phenotype) were compared to controls (shown as insets to figures). Next, data from the outcome-dependent P-DIE and P-SUR cohorts were compared.

#### Midbrain

**Fig. 3:**
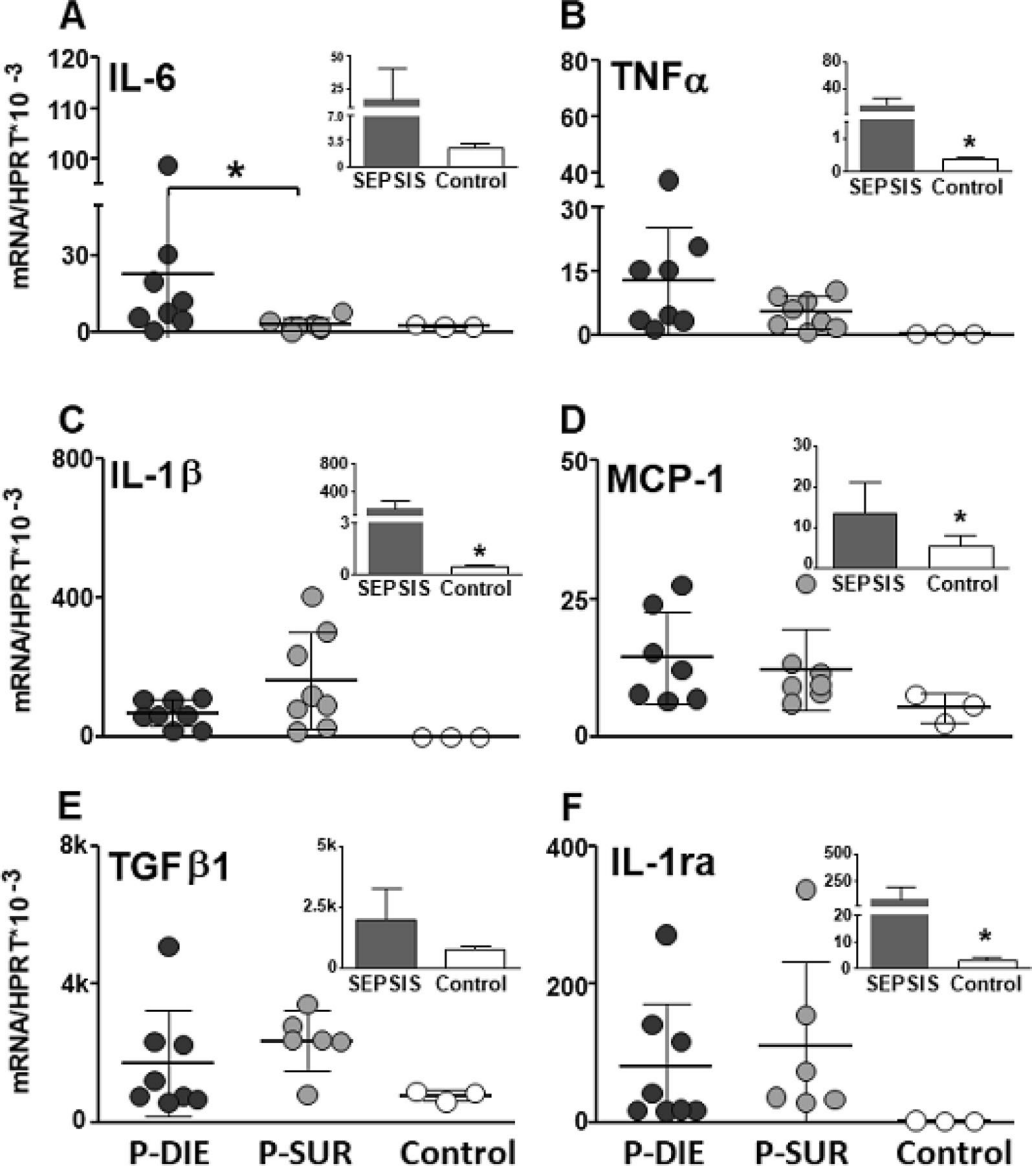
Comparison of cytokine gene expression in the midbrain of septic (CLP) mice predicted-to-die (P-DIE), predicted-to-survive (P-SUR) and healthy control mice. P-DIE mice were sacrificed within days 2–4 post-CLP and matched with P-SUR mice. Insets depict comparison of all pooled septic mice (regardless of the predicted outcome) to healthy controls. *k* = 1000 ×

Generally, there was a significant increase in the mRNA expression in the 4/6 analysed cytokines (i.e., TNFα, IL-1β, MCP-1 and IL-1ra) (Fig. 3). Of those cytokines, only IL-6 expression was further exacerbated (by > sevenfold) in P-DIE compared to P-SUR mice (*p* < 0.05).

#### Cerebellum

**Fig. 4:**
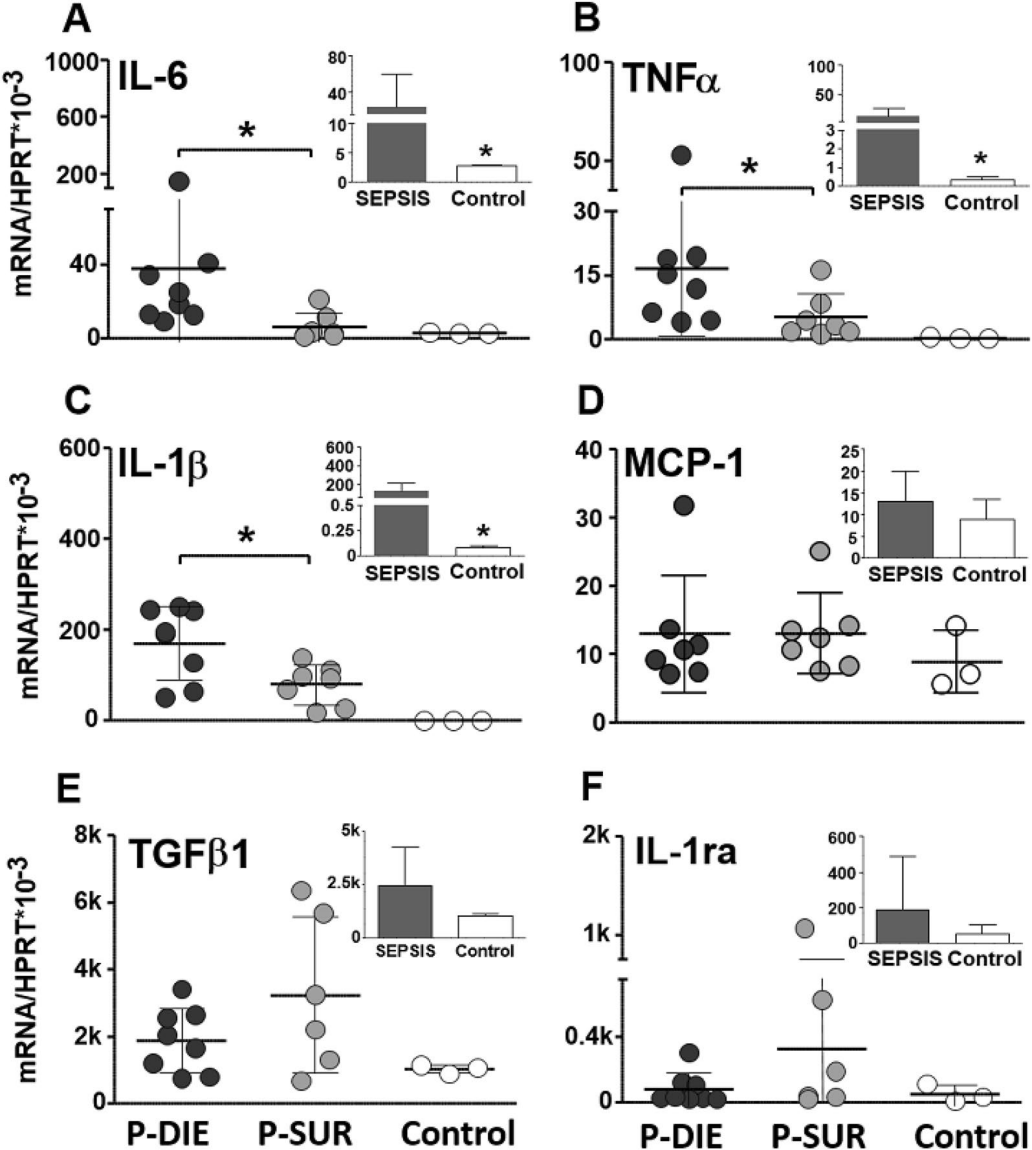
Comparison of cytokine gene expression in the cerebellum of septic (CLP) mice predicted-to-die (P-DIE), predicted-to-survive (P-SUR) and healthy control mice. P-DIE mice were sacrificed within days 2–4 post-CLP and matched with P-SUR mice. Insets depict comparison of all pooled septic mice (regardless of the predicted outcome) to healthy controls. *k* = 1000 ×

Overall, sepsis strongly enhanced the IL-6 (> eightfold), TNFα (> 32-fold) and IL-1β (25-fold) gene expression (insets in Fig. 4). The expression of the same three cytokines was higher by > 6, > 3 and > twofold (respectively; *p* < 0.05) in P-DIE compared to P-SUR animals (Fig. 4).

#### Hypothalamus

**Fig. 5:**
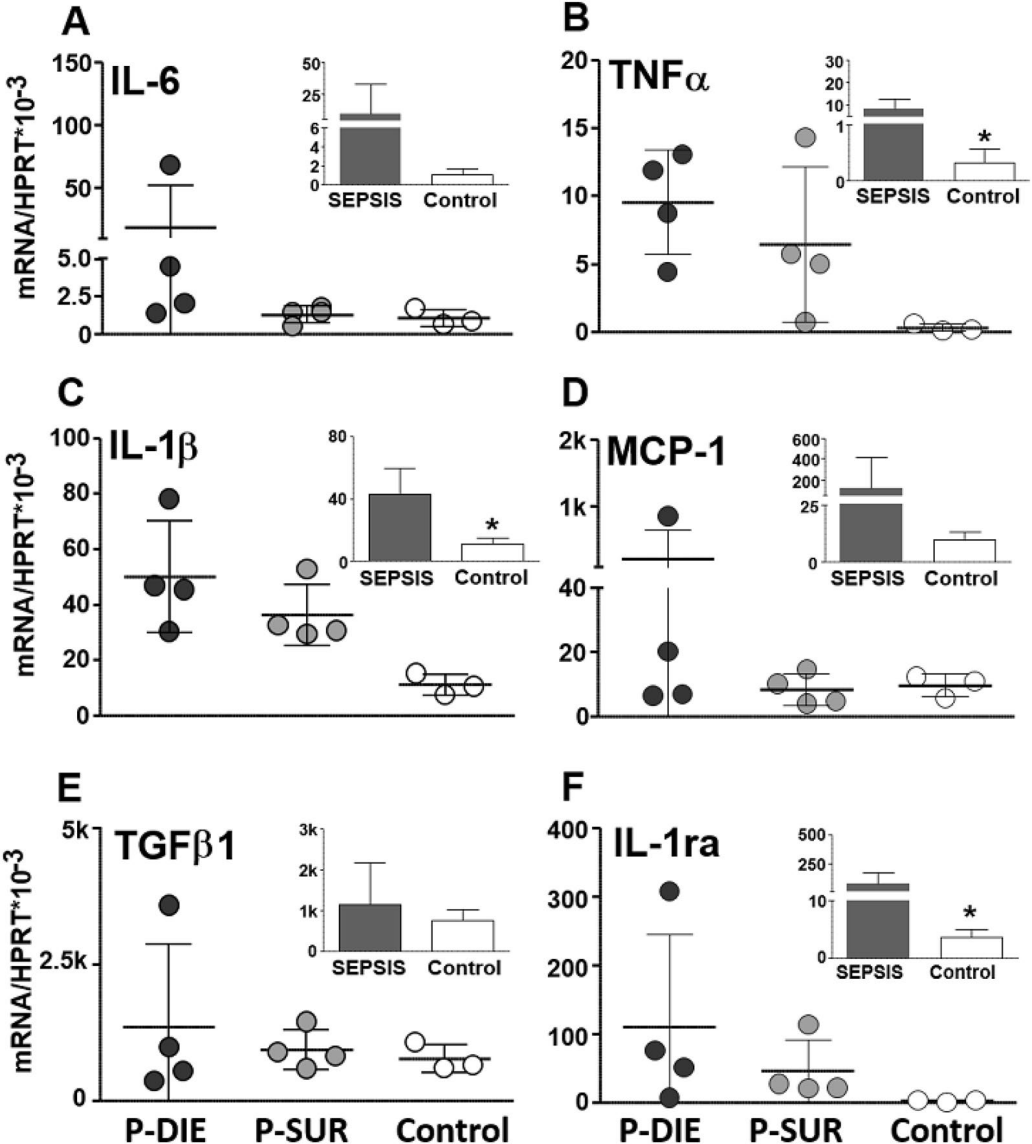
Comparison of cytokine gene expression in the hypothalamus of septic (CLP) mice predicted-to-die (P-DIE), predicted-to-survive (P-SUR) and healthy control mice. P-DIE mice were sacrificed within days 2–4 post-CLP and matched with P-SUR mice. Insets depict comparison of all pooled septic mice (regardless of the predicted outcome) to healthy controls. *k* = 1000 ×

Overall, CLP sepsis strongly enhanced the IL-6 (> sixfold) (TNFα (> 23-fold), IL-1β (> twofold), IL-1ra (> 22-fold) MCP-1 (> 13-fold) gene expression in the hypothalamus (insets in Fig. 5). However, there were no further differences between P-DIE and P-SUR mice (Fig. 5).

#### Hippocampus

**Fig. 6:**
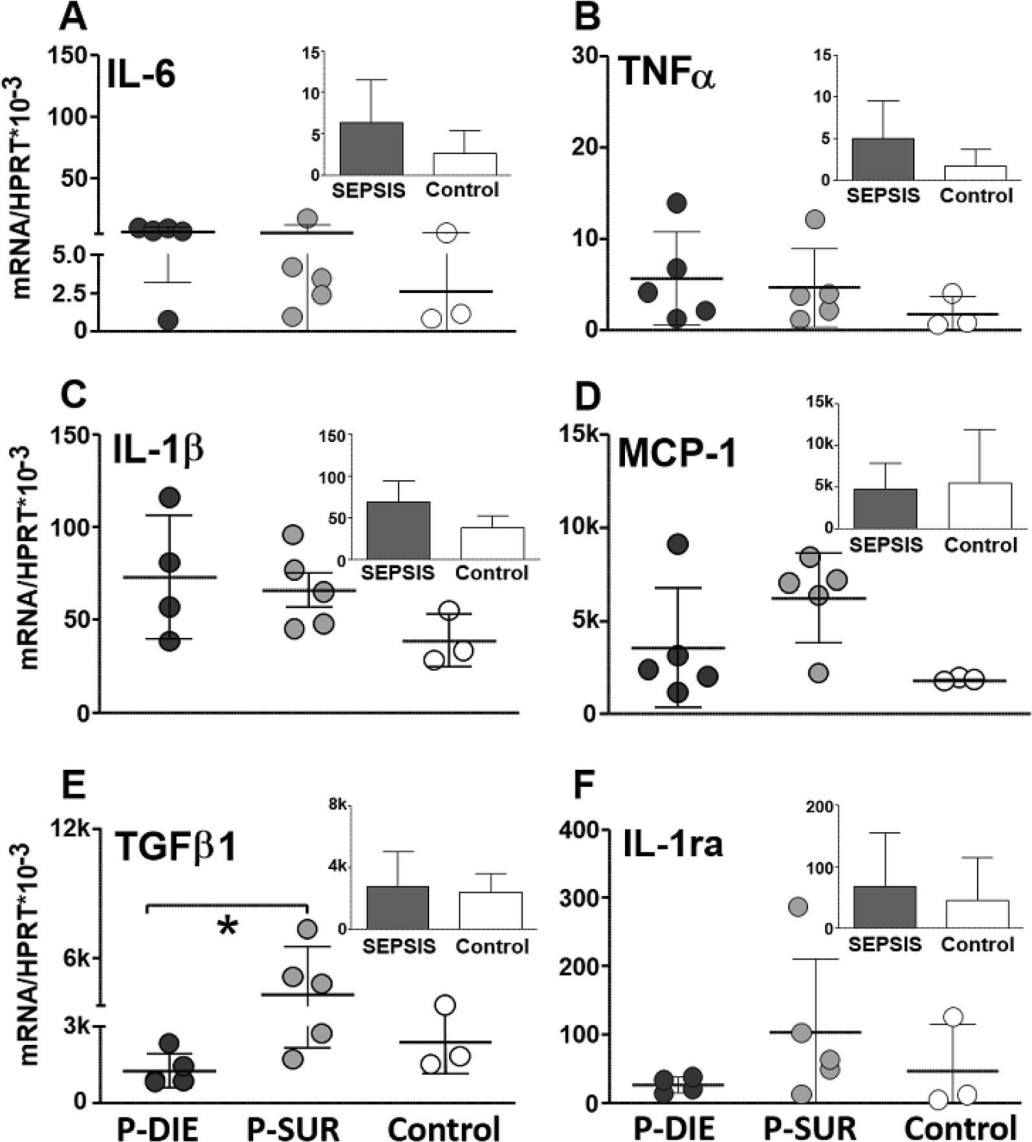
Comparison of cytokine gene expression in the hippocampus of septic (CLP) mice predicted-to-die (P-DIE), predicted-to-survive (P-SUR) and healthy control mice. P-DIE mice were sacrificed within days 2–4 post-CLP and matched with P-SUR mice. Insets depict comparison of all pooled septic mice (regardless of the predicted outcome) to healthy controls. *k* = 1000 ×

Overall, CLP sepsis failed to alter the gene expression in any of the investigated cytokines (insets in Fig. 6). The only outcome-based comparison revealed a 70% decrease of the TGFβ1 in P-DIE versus P-SUR mice (Fig. 6).

### Activation of astrocytes and microglial cells was unaffected by CLP phenotypes

The standard HE examination did not reveal any marked differences between septic and healthy mice (data not shown) regarding the tissue damage and/or infiltration of inflammatory cells such as neutrophils and/or lymphocytes. Myelinophages, indicating mobile resorption due to demyelination, were not evident. There was an enhanced vacuolization of septic brains (irrespective of outcome) across all regions except neocortex and hippocampus; similar vacuolization albeit less frequent was also present in control mice (Supplementary Table 1).

A more specific immunohistochemical analysis revealed several findings. Detailed comparison across brain regions and between specified mouse groups are listed in Tables 1 and 2.

**Fig. 7:**
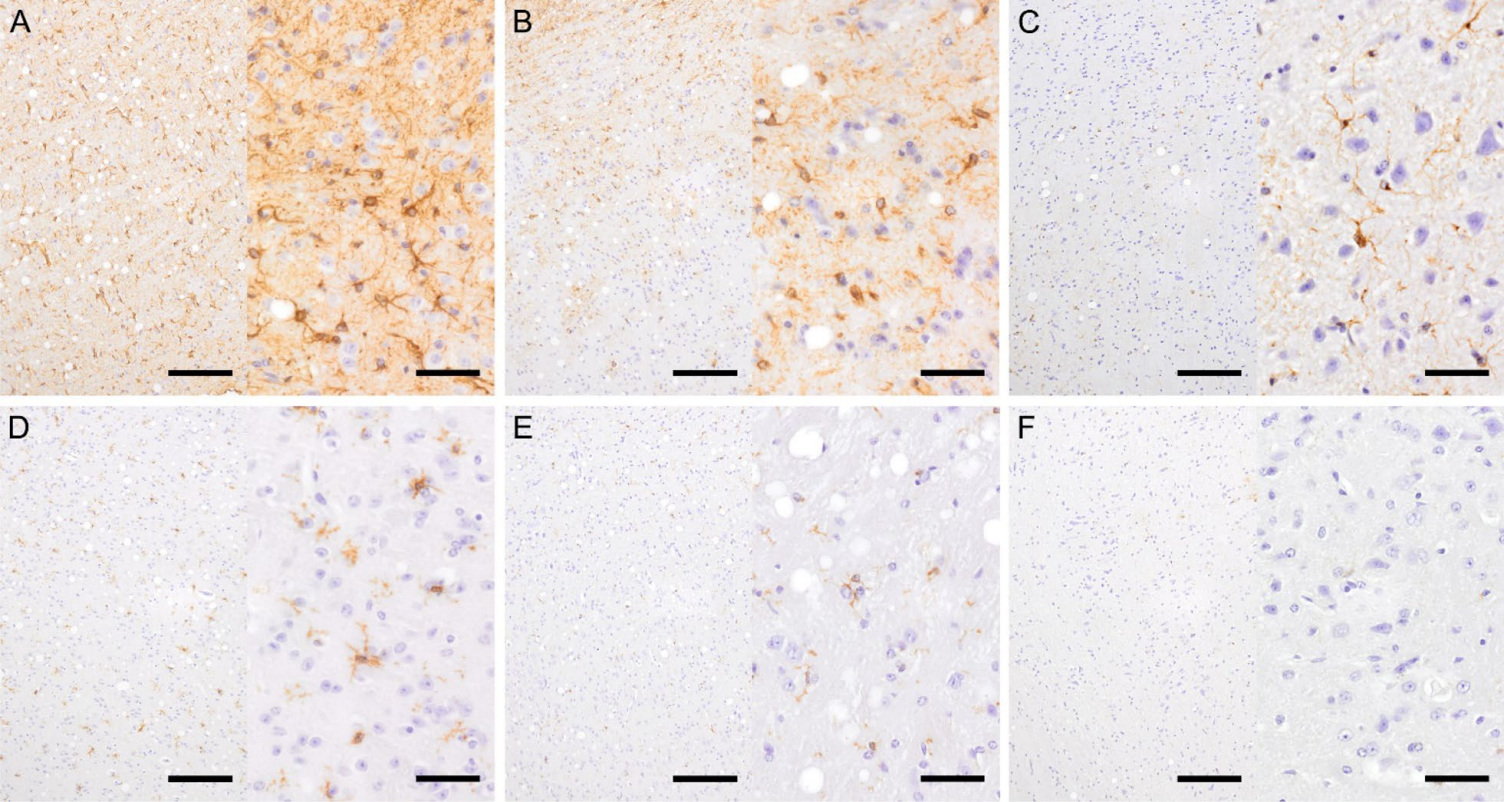
Low and high-resolution images of immunohistochemical staining with GFAP- (**A**–**C**) and IBA-1 (**E**, **F**) antibodies in the medulla oblongata of septic P-DIE mice (**A**, **D**), P-SUR mice (**B**, **E**) and CON mice (**C**, **F**). bar = 160 µm for low resolution images; bar = 40 µm for high resolution images; *P-DIE* predicted-to-die, *P-SUR* predicted-to-survive, *IBA-1* ionized calcium-binding protein (microglia/macrophage-specific), *GFAP* glial fibrillary acidic protein (astrocyte-specific)

GFAP staining showed a widespread increase in the astrocyte activity in septic mice (regardless of outcome) compared to healthy controls; it was most pronounced in the cerebellum. The outcome-based comparison demonstrated an exacerbated astrocyte activation only in the medulla oblongata of P-DIE mice versus P-SUR (Table 1, Fig. 7A–C).

IBA-1 staining demonstrated a strong generalized exacerbation of sepsis-induced macrophage activity in the neocortex, cerebellum, medulla oblongata, and midbrain, but not in the remaining regions (Table 2). Outcome-dependent stratification failed to reveal any apparent difference in the microglial activity including medulla oblongata (Fig. 7D–F).

HE and FluoroJade B staining did not reveal any apparent neuronal death in the examined brain regions (data not shown).

### Blood–brain barrier permeability was unaffected by CLP phenotypes

Finally, we examined the integrity of the BBB in the midbrain, cerebellum and neocortex of septic mice in the early phase of the disease.

#### Sodium fluorescein permeability

**Fig. 8:**
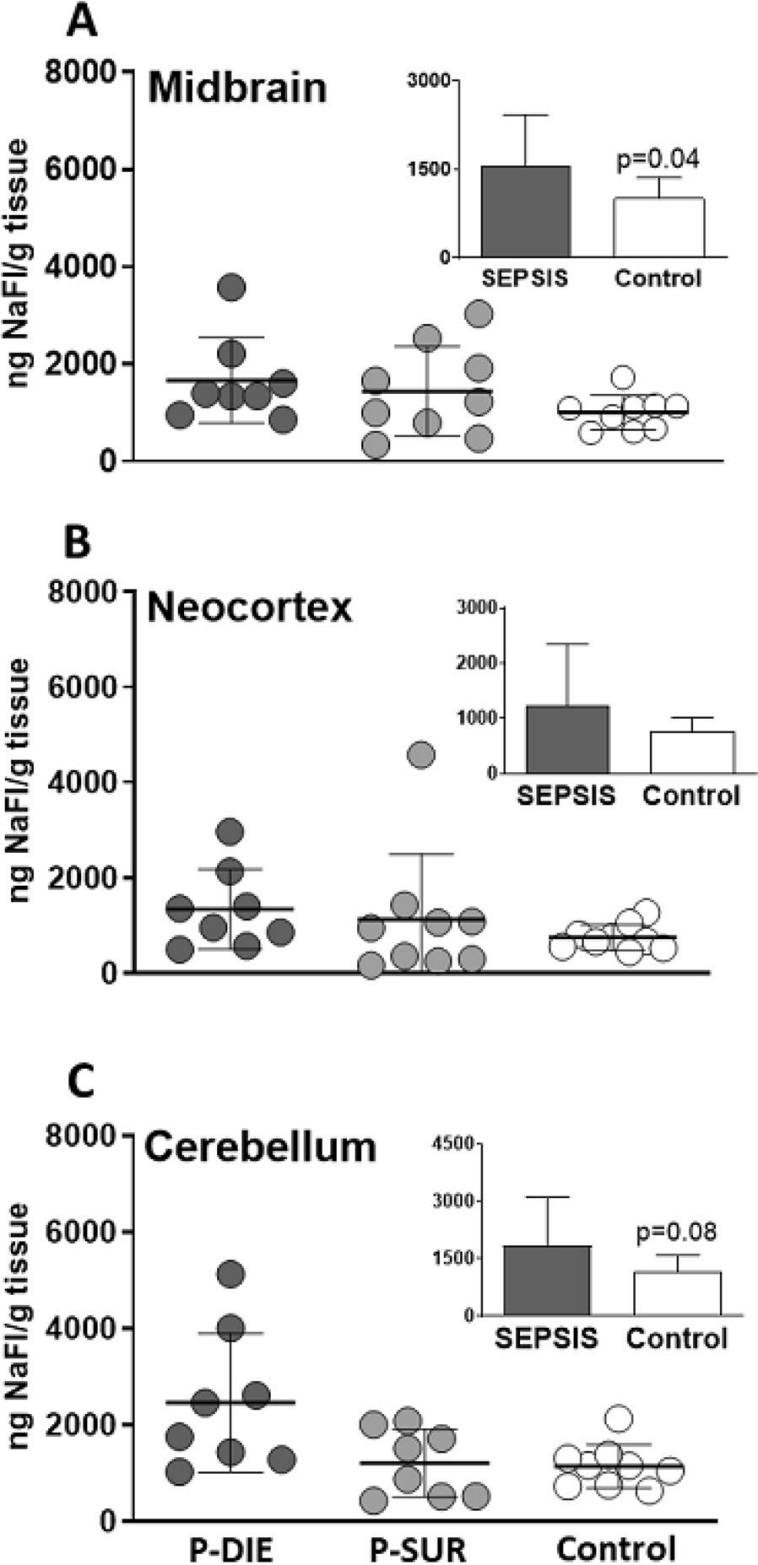
Comparison of blood–brain barrier (BBB) permeability with sodium-fluorescein (NaFl) in the midbrain (**A**), neocortex (**B**) and cerebellum (**C**) of septic P-DIE mice, P-SUR mice and healthy control mice. P-DIE mice were sacrificed within days 2–4 post-CLP and matched with P-SUR mice. *P-DIE* predicted-to-die, *P-SUR* predicted-to-survive. Insets depict comparison of all pooled septic mice (regardless of the predicted outcome) to healthy controls

Comparison between the P-DIE and P-SUR subgroups (and each individual group to control) showed similar fluorescein permeability in all three tested brain regions (Fig. 8). Fluorescence microscopy showed a strong signal within capillaries in most P-DIE and P-SUR mice injected with NaFl, but this fluorescence was never detected outside capillary vessels (Supplementary Fig. 3). In pooled septic mice, CLP marginally modulated the fluorescein signal in the midbrain but not in other two regions compared to control (insets in Fig. 8).

We also repeated the assay in a separate group of male mice for the same three brain regions. The fluorescein permeability between P-DIE and P-SUR mice was similar in all three regions and individually no different from control (Supplementary Fig. 4). Only in pooled CLP mice compared to control, the fluorescein intensity was higher.

#### *Electron* microscopy

**Fig. 9:**
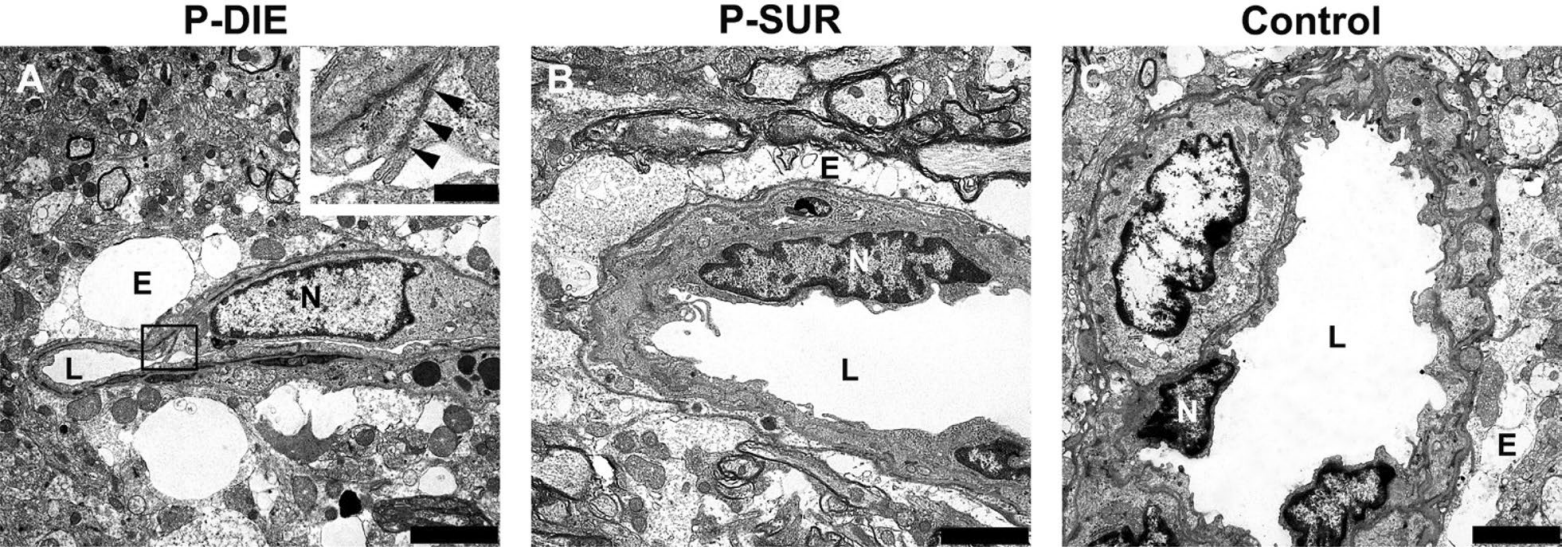
Representative images of the thalamus of septic P-DIE (**A**), P-SUR (**B**) and healthy control (**C**) mice in transmission electron microscopy (TEM). No lesions in cellular ultrastructure were detectable in any of the evaluated animals regardless of group assignment. The blood–brain barrier (BBB) was intact even in animals of the P-DIE group. The insert shows the magnification of an intact tight junction (arrowheads) annotated by a rectangle (**A**). Note mild perivascular edema (**E**) present in all groups. Bars = 2500 nm, bar in insert = 500 nm; *E* perivascular edema, *L* capillary lumen, *N* endotheliocyte nucleus, *P-DIE* predicted-to-die, *P-SUR* predicted-to-survive

In the second step, we examined cell ultrastructure by TEM in all seven dissected brain regions. Specifically, the integrity of the lamina basalis as well as composition and structure of the tight junctions forming the BBB were evaluated. In all examined brain tissue specimens from septic mice (regardless of outcome), cellular structures were intact (Fig. 9).

## Discussion

Sepsis-associated encephalopathy (SAE) is a poorly understood condition that is associated with decreased survival [2]. While the existing studies suggest a deregulated neuronal function during sepsis, clear-cut evidence of pathophysiological derangements in the CNS, their grade, dynamics and their association with sepsis outcomes are lacking. In our mechanistic study, CLP mice were stratified into early-phase lethal (P-DIE) and surviving (P-SUR) phenotypes and juxtaposed with non-septic controls. Best to our knowledge, such an outcome-based separation was done for the first time for the pre-clinical CNS analysis; it enabled a more in-depth mechanistic characterization of the CNS pathophysiology by isolating specific death/life signals in two distinct septic cohorts (rather than a simple comparison of healthy and septic subjects).

Regardless of outcome, CLP mice showed a consistent rise of serotonin, HIAA and HVA in nearly all brain regions. There is a demonstrated relationship between the development of SAE and changes in neurotransmitter release/concentrations including acetylcholine [49], dopamine [50], serotonin [51] and norepinephrine [52]. Hippocampal serotonin increased in endotoxemic BALB/c and C57BL/6J mice [53], while brain and plasma tryptophan (a substrate for serotonin synthesis) was elevated in septic rats [54]. Moreover, it has been shown that the serotonin precursor, 5-HIAA and the 5-HIAA/serotonin ratio increased in most tissues in non-severe septic animals, [51, 55] and declined with the progression of severe sepsis [54].

A phenotype-based analysis of septic mice revealed several findings. First, P-DIE mice showed rise of HVA, dopamine and DOPAC in the hippocampus, hypothalamus and medulla compared to P-SUR. In the brain, dopamine is critical for the maintenance of working memory and the regulation of emotion [56] and its overdose is associated with the development of SAE [57]. Yet, evidence regarding dopamine activity/fluctuations in sepsis is inconsistent. One early CLP study reported high dopamine level in the hippocampus, striatum and medulla (along with low levels of HVA and 3MT metabolites), suggesting a decreased turnover of dopamine in sepsis [51]. Another similar experiment demonstrated that dying CLP rats with encephalopathy exhibited lower dopamine concentration compared with non-SAEs rats [54]. A newer study showed an increase in brain dopamine turnover and dopaminergic activity during sepsis [50]. Recent research suggests that administration of a small dose of l-dopamine in early sepsis limited neuroinflammation, improved neuroplasticity and reversed sepsis-induced decrease in hippocampal dopamine [58]. In comparison to sepsis-induced neuroinflammation, lack of DRD3 dopaminergic signaling in LPS-induced mouse neuroinflammation, increased the expression of the anti-inflammatory mediator Fizz1 in glial cells and increased M1-to-M2 ratio, eventually alleviating neuroinflammation [59]. In our study, amine neurotransmitters in P-DIE mice were increased compared to P-SUR mice but these observations should be reproduced and associated with behavioral/cognitive endpoints.

We also examined a regional inflammation activation in the brain. Regardless of the predicted outcome, sepsis increased cytokine tissue expression. Surprisingly, The TNFα and IL-6 expression was higher in P-DIE than in P-SUR only in cerebellum, and IL-6 was higher only in the midbrain. TNF-*α,* IL-1β and IL-6 belong to the most important systemic pro-inflammatory factors undergoing activation at the onset of sepsis. Robust inflammation severely impairs dopamine, norepinephrine and serotonergic neurotransmission in the CNS leading to a cognitive decline [50, 60]. Inversely, neurotransmitter dysregulation can frequently promote inflammation. Nolan et al*.* recently showed that dopamine activated the NF-κB pathway in macrophages and primed the NLRP3 inflammasome, inducing cytokine production [61]. IL-1β secreted by activated microglia can suppress axonal development and synapse formation through activation of the p38-MAPK signaling pathway associated with memory impairments in septic patients [62].

We next sought to confirm the robust genomic inflammatory activation on the cellular level. Sepsis caused a robust activation of astrocytes and microglia in the brain but this effect was regionalized and outcome independent. Compared to P-SUR, only astrocyte activation in the medulla oblongata of P-DIE mice was further exacerbated; we currently cannot interpret the role of that particular observation. It is clear that astrocytes constitute the principal homeostatic cells of the CNS contributing to the brain defense against infection and regulating brain immune response and medulla itself contributes to regulating circulation and respiration [20]. It was shown that mitochondrial biogenesis was significantly elevated in astrocytes (via the IL-6/AMPK pathway) to meet the high-energy demand and recover the ultrastructure of the mitochondria [63].

Microglia play an even more direct defensive role in response to pathogens and injury [20, 64]. Their robust upregulation in the hippocampus results in learning and memory impairment [65]. Michels et al*.* demonstrated that inhibition of microglia can reverse exacerbation of cerebral and systemic inflammation during the development of severe sepsis in male rats and improved long-term cognitive performance in sepsis survivors [66]. Microglial cells, acting as antigen-presenting cells, express a variety of receptors, such as TLRs, major histocompatibility complex, CX3CR1 chemokine receptor and CD11b/CD45 [67]. Therefore, LPS, other pathogen components and inflammation in the peripheral blood can activate microglia when they pass through the increased permeability of the BBB [68].

Interestingly, the lethal sepsis phenotype (versus survivors) did not affect the BBB permeability in the examined regions. Absence of neutrophil and/or lymphocyte infiltrates in the brain is consistent with that finding. BBB dysfunction has been considered one of the key factors in promoting SAE in sepsis [21]. Given that BBB disruption is uniformly present in LPS endotoxemia [69], this phenomenon has been also assumed in sepsis but data are far less consistent regarding the latter. LPS does not properly recapitulate sepsis and endotoxemia is not recommended in sepsis modeling [31]. In line with our findings, Pang et al*.* recently showed that CLP mice displayed axonal injury, robust microglial activation and cytotoxic edema in the cortex, thalamus and hippocampus but the BBB integrity was intact [70]. Species differences in BBB resistance are possible given that acute peritonitis in the rat led to an apparent BBB leakage [71].

Our study has several limitations. First, only the early phase of sepsis was evaluated (days 1–4 post-CLP), therefore, it is unclear to what extent the recorded differences relate to the long-term cognitive outcomes (i.e., in P-SUR mice). Second, we did not perform any comparative behavioral and/or cognitive testing, thus, we were unable to define the direct translation of the observed CNS derangements into the cognitive behavior. Third, we only used female sex and did not measure estrogen and/or characterize the estrus cycle in female mice to juxtapose it with the observed CNS changes. Fourth, we recognize that the ambient temperature used in our study (21–23 °C; below the thermoneutral zone for mice) may have induced cold stress, potentially impacting the physiological responses of septic mice [72, 73]. Had this study been conducted in the thermoneutral conditions, it is likely that the current BT-based thresholds we used for phenotype stratification would not have been applicable due to differential thermoregulation processes in the mouse exposed to the non-versus thermoneutral temperatures [74]. Given that this design shortcoming has clear translational implications [75], it should be carefully addressed on the level of good scientific practice in preclinical (translational) sepsis research such as MQTiPSS. Lastly, we did not use aged but mature animals and it is plausible that aged animals display more robust neurotransmitter and inflammatory CNS alterations.

## Conclusions

In this mechanistic study, we characterized lethal and surviving phenotypes in abdominal mouse sepsis using outcome stratification. The phenotypic comparison did not demonstrate clear-cut conclusions. While the lethal phenotype (compared to survivors) was associated with a stronger deregulation of the regional brain amine metabolism, the inflammatory activation was relatively uniformly widespread with only limited outcome-dependent changes. Interestingly, the lethal sepsis phenotype was not associated with an enhanced BBB permeability (compared to sepsis survivors). Further research of SAE pathogenesis is warranted, especially in aged animals and in conjunction with behavioral and/or cognitive tests.

## Supplementary Information

Supplementary Material 1.

## Data Availability

The datasets generated and analyzed during the current study are available from the corresponding author on reasonable request.

## Abbreviations

BBB: Blood–brain barrier
BT: Body temperature
cDNA: Complementary DNA
CIP: Critical illness polyneuropathy
CIM: Critical illness myopathy
CLP: Cecal ligation and puncture
CNS: Central nervous system
CON: Healthy controls
DA: Dopamine
DOPAC: 3,4-Dihydroxyphenylacetic acid
ELMI: Electron microscopy
GFAP: Glial fibrillary acidic protein
HE: Hematoxylin–Eosin
HPRT: Hypoxanthine–guanine phosphoribosyltransferase
HIAA: Hydroxyindoleatic acid
HVA: Homovanillic acid
IBA-1: Ionized calcium-binding adaptor molecule 1
ICU: Intensive care unit
IL-6: Interleukin-6
IL-1β: Interleukin-1β
IL-1ra: Interleukin-1 receptor antagonist
LC–MS/MS: Liquid chromatography tandem mass spectrometry assay
l-DOPA: Dihydroxy-l-phenylalanine
LLOQ: Lower limit of quantification
M-CASS: Mouse clinical assessment scoring system
MCP-1: Monocyte chemotactic protein -1
MHGP: Methoxy-hydroxy-phenylethylen glycol
NaFl: Sodium-fluorescein
NE: Norepinephrine
PBS: Physiologically buffered saline
P-DIE: Predicted-to-die
P-SUR: Predicted-to-survive
(q)SOFA score: Quick Sequential Organ Failure Assessment Score
Real-time PCR: Real-time polymerase chain reaction
5-HT: Serotonine
SAD: Sepsis-associated delirium
SAE: Sepsis-associated encephalopathy
SOFA score: Sequential Organ Failure Assessment Score
TGF-β1: Transforming growth factor-β1
TNFα: Tumor necrosis factor alpha
