# Supplementary material for: Lethal versus surviving sepsis phenotypes displayed a partly differential regional expression of neurotransmitters and inflammation and did not modify the blood–brain barrier permeability in female CLP mice
Source: Intensive Care Med Exp. 2024 Nov 4;12:96. doi: 10.1186/s40635-024-00688-7 (PMC11535104; doi:10.1186/s40635-024-00688-7)
Supplement: Supplementary file 1 — Supplementary Material 1. [file 40635_2024_688_MOESM1_ESM.docx]

**Supplementary Materials**

**Supplementary Table 1. Vacuolization in all evaluated brain regions.**

|  | **CON** | **P-SUR** | **P-DIE** | **CON/P-SUR** | **CON/P-DIE** | **P-SUR/P-DIE** |
| --- | --- | --- | --- | --- | --- | --- |
| Brain region | n = 3 | n = 8 | n = 8 | p-values | p-values | p-values |
| neocortex | 0.0 (0.0-0.0) | 0.0 (0.0-0.8) | 0.0 (0.0-0.0) | 0.36 | 1.00 | 0.14 |
| cerebellum | 2.0 (2.0-2.0) | 3.0 (2.0-3.0) | 3.0 (2.0-3.0) | 0.08 | 0.08 | 1.00 |
| medulla oblongata | 1.0 (1.0-1.0) | 2.0 (2.0-2.0) | 2.0 (2.0-2.0) | 0.03 | 0.02 | 0.32 |
| hypothalamus | 0.0 (0.0-0.0) | 2.0 (1.0-2.0) | 1.0 (0.3-2.0) | 0.02 | 0.05 | 0.33 |
| midbrain | 1.0 (0.0-1.0) | 2.0 (2.0-2.0) | 2.0 (2.0-2.0) | 0.02 | 0.07 | 0.32 |
| thalamic nuclei | 0.0 (0.0-0.0) | 1.0 (0.0-1.0) | 1.0 (0,0-1,8) | 0.09 | 0.09 | 0.78 |
| hippocampus | 1.0 (0.0-1.0) | 1.0 (0.0-1.0) | 1.0 (0,0-1.0) | 0.66 | 0.66 | 1.00 |
| striatum | 0.0 (0.0-0.0) | 0.5 (0.5-1.0) | 1.0 (0.0-2.0) | 0.14 | 0.09 | 0.26 |

p<0.05 by Mann-Whitney-U-test (with an adjustment for multiple comparisons).HE staining evaluation: 0 no vacuolisation, 1 mild vacuolisation, 2 moderate vacuolization, 3 severe vacuolisation


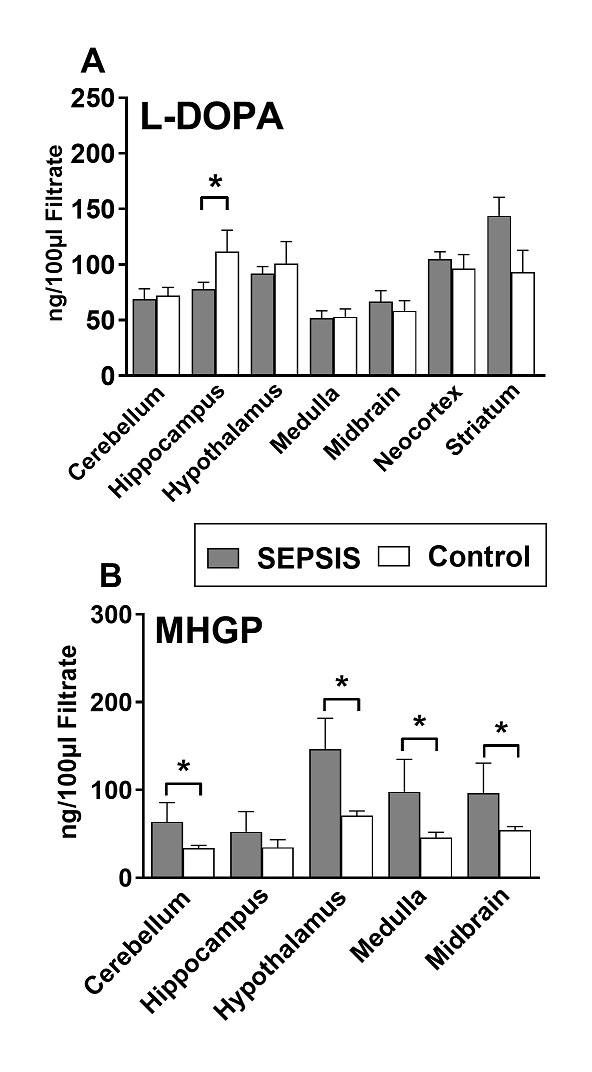


**Supplementary Figure 1.** Regional concentrations of L-DOPA (A; dopamine precursor) and MHGP (B; principal norepinephrine metabolite) in the brains of septic (CLP) and healthy control mice. Septic mouse data were pooled regardless of the predicted outcome. Mice were sacrificed within days 2-4 post-CLP. In L-DOPA: n=10/sepsis group; n=4/control (each region). In MHGP: n=6/sepsis group; n=3/control (each region). Data as mean ± SD.  **P* < 0.05. L-DOPA: levodopa, l-3,4-dihydroxyphenylalanin; MHGP: 3-methoxy-4-hydroxyphenylglycol.


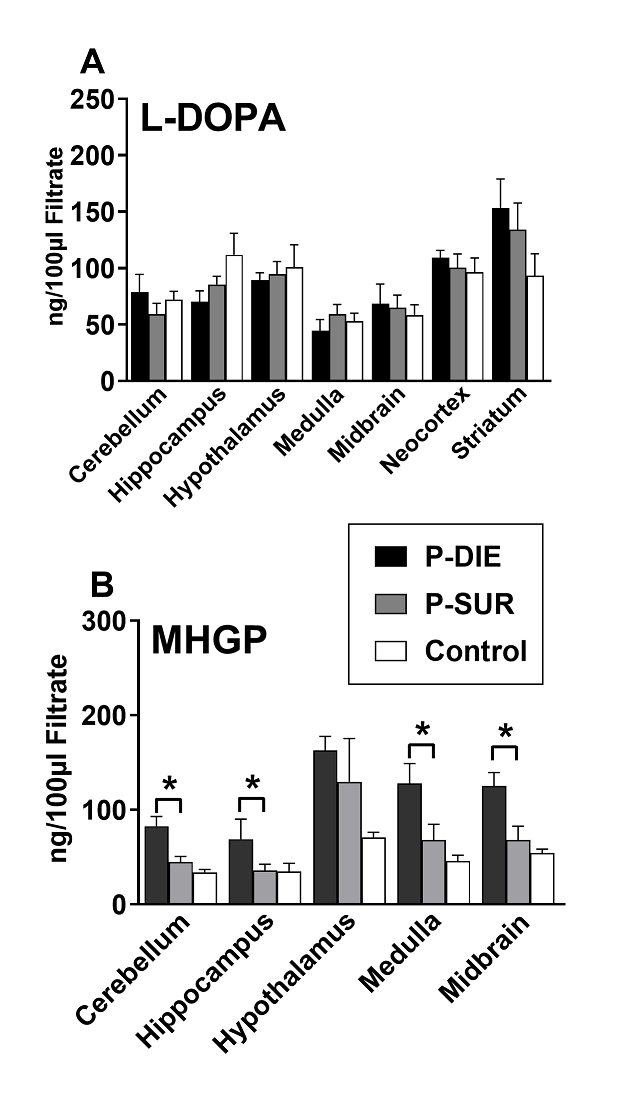


**Supplementary Figure 2.** Regional concentrations of L-DOPA (A; dopamine precursor) and MHGP (B; principal norepinephrine metabolite) in the brains of septic (CLP) mice predicted-to-die (P-DIE), predicted-to-survive (P-SUR) and healthy control mice. Mice were sacrificed within days 2-4 post-CLP. In L-DOPA: n=5/P-DIE and P-SUR group each; n=4/control (each region). In MHGP: n=3/each group (each region). Data as mean ± SD.  **P* < 0.05. L-DOPA: levodopa, l-3,4-dihydroxyphenylalanin; MHGP: 3-methoxy-4-hydroxyphenylglycol.


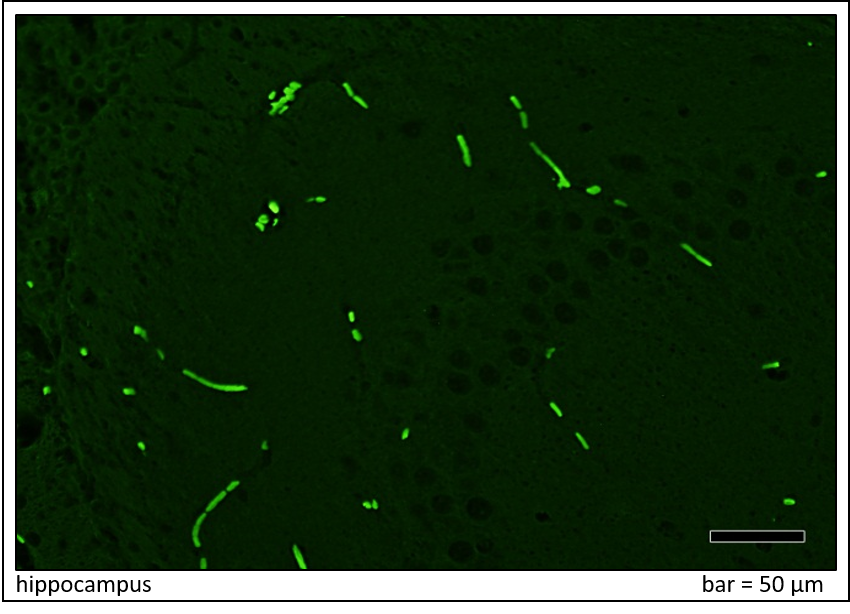


**Supplementary Figure 3.** Fluorescence microscope image (a representative example): an intravascular fluorescence within capillary vessels of hippocampus harvested from a surviving (P-SUR) mouse. The shown signal originates from an intravenous injection of sodium fluorescein (NaFl, 200 ml of 0.5%; see Material and Methods) prior to brain harvesting to assess the blood brain barrier integrity.

**
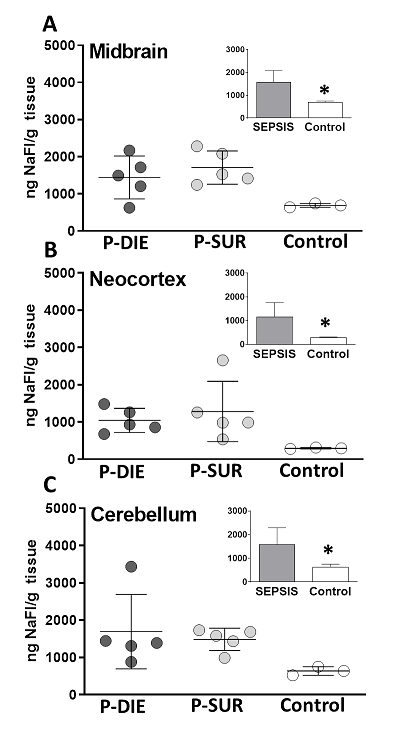
**

**Supplementary Figure 4.** Comparison of Blood Brain Barrier (BBB) permeability with sodium-fluorescein (NaFl) in the midbrain (A), neocortex (B) and cerebellum (C) of male (three-month-old, CD-1) septic P-DIE mice, P-SUR mice and healthy control mice. P-DIE mice were sacrificed within days 2-4 post-CLP and matched with P-SUR mice. P-DIE: predicted-to-die; P-SUR: predicted-to-survive. Insets depict comparison of all pooled male septic mice (regardless of the predicted outcome) to healthy controls. Data as mean ± SD.  **P* < 0.05.
